# On the additive artificial intelligence-based discovery of nanoparticle neurodegenerative disease drug delivery systems

**DOI:** 10.3762/bjnano.15.47

**Published:** 2024-05-15

**Authors:** Shan He, Julen Segura Abarrategi, Harbil Bediaga, Sonia Arrasate, Humberto González-Díaz

**Affiliations:** 1 Department of Organic and Inorganic Chemistry, University of Basque Country UPV/EHU, 48940 Leioa, Spain; 2 IKERDATA S.L., ZITEK, UPV/EHU, Rectorate Building, nº6, 48940 Leioa, Greater Bilbao, Basque Country, Spain; 3 Painting Department, Fine Arts Faculty, University of the Basque Country UPV/EHU, 48940, Leioa, Biscay, Basque Country, Spain; 4 Instituto Biofisika (UPV/EHU-CSIC), 48940 Leioa, Spain; 5 IKERBASQUE, Basque Foundation for Science, 48011 Bilbao, Biscay, Spain

**Keywords:** artificial neural network (ANN), linear discriminant analysis (LDA), machine learning, nanoparticle, neurodegenerative diseases

## Abstract

Neurodegenerative diseases are characterized by slowly progressing neuronal cell death. Conventional drug treatment strategies often fail because of poor solubility, low bioavailability, and the inability of the drugs to effectively cross the blood–brain barrier. Therefore, the development of new neurodegenerative disease drugs (NDDs) requires immediate attention. Nanoparticle (NP) systems are of increasing interest for transporting NDDs to the central nervous system. However, discovering effective nanoparticle neuronal disease drug delivery systems (N2D3Ss) is challenging because of the vast number of combinations of NP and NDD compounds, as well as the various assays involved. Artificial intelligence/machine learning (AI/ML) algorithms have the potential to accelerate this process by predicting the most promising NDD and NP candidates for assaying. Nevertheless, the relatively limited amount of reported data on N2D3S activity compared to assayed NDDs makes AI/ML analysis challenging. In this work, the IFPTML technique, which combines information fusion (IF), perturbation theory (PT), and machine learning (ML), was employed to address this challenge. Initially, we conducted the fusion into a unified dataset comprising 4403 NDD assays from ChEMBL and 260 NP cytotoxicity assays from journal articles. Through a resampling process, three new working datasets were generated, each containing 500,000 cases. We utilized linear discriminant analysis (LDA) along with artificial neural network (ANN) algorithms, such as multilayer perceptron (MLP) and deep learning networks (DLN), to construct linear and non-linear IFPTML models. The IFPTML-LDA models exhibited sensitivity (Sn) and specificity (Sp) values in the range of 70% to 73% (>375,000 training cases) and 70% to 80% (>125,000 validation cases), respectively. In contrast, the IFPTML-MLP and IFPTML-DLN achieved Sn and Sp values in the range of 85% to 86% for both training and validation series. Additionally, IFPTML-ANN models showed an area under the receiver operating curve (AUROC) of approximately 0.93 to 0.95. These results indicate that the IFPTML models could serve as valuable tools in the design of drug delivery systems for neurosciences.

## Introduction

Over time, there has been a significant shift in global dietary habits and lifestyle standards. Poor dietary choices, irregular eating patterns, extended working hours, and sedentary behaviors have contributed to a trend towards an unhealthy lifestyle [1]. This shift has resulted in a rise in chronic degenerative diseases among the elderly population. These diseases encompass a diverse range of conditions characterized by the gradual deterioration of bodily structures and functions [2–3]. Although the exact causes leading to these diseases remain unidentified, there is evidence that oxidative damage plays a crucial role in the progressive neuronal cell death, particularly through the generation of reactive oxygen and nitrogen species [4–5]. In this regard, Alzheimer’s and Parkinson’s diseases are the most severe and untreatable conditions. Conventional drug treatment methods, such as acetylcholinesterase inhibitor drugs, often encounter obstacles due to their inadequate solubility, limited bioavailability, and inability to effectively penetrate the blood–brain barrier (BBB) [6]. Therefore, there is an urgent need to focus on the advancement of novel neurodegenerative disease drugs (NDDs) [7–8]. The major obstacle encountered by NDDs is the selectivity of the BBB, which limits the number of therapeutic substances able to reach the brain in order to induce a positive effect. Recently, many efforts have been made to develop systems that facilitate the passage of NDDs through the BBB.

Interestingly, nanoparticle (NP) systems are gaining increasing interest among the possible nanomedicine strategies for NDD transport to the central nervous system (CNS) [9–10]. For simplicity, we are going to call them nanoparticle neuronal diseases drug delivery systems (N2D3Ss). N2D3Ss have the ability to protect NDDs from chemical and enzymatic degradation, direct the active compound towards the target site with a substantial reduction of toxicity for the adjacent tissues, and help the NDDs to pass physiological barriers, increasing bioavailability without resorting to high dosages [5,11]. Therefore, researchers are studying and developing new treatment approaches that use N2D3Ss for diagnosis and treatment [12–15].

Also, over the last few years, artificial intelligence/machine learning (AI/ML) models have been applied successfully to solve problems in different disciplines, especially in the interface of chemistry and ND research [16–19]. In this regard, we consider AI/ML to be helpful in the development of N2D3Ss to select the most efficient combination of NP and drug, taking into account properties regarding chemical absorption, distribution, metabolism, excretion, and toxicity (ADMET), and the biological activity regarding NDs [20]. Nevertheless, there is relatively limited experimental data on NPs reported in the scientific literature in comparison to drugs, which increases the difficulty of designing systems based on AI/ML techniques.

An additional essential downside of developing N2D3Ss with AI/ML techniques is the great complexity of the data to be explored. As a result, N2D3S development by the additive approach requires an AI/ML technique to achieve multioutput and multilabel classification [21–24]. In addition, the AI/ML technique includes a pre-processing step to perform information fusion (IF) of the preclinical NDD assay and NP cytotoxicity datasets. Nevertheless, most of the AI/ML methods reported to date only consider the structural/molecular descriptors of the NDDs or NPs as input. Therefore, these methods exclude completely non-structural parameters, specifically experimental conditions of the assays, in order to list NDD or NP labels. Consequently, the resulting model cannot predict multioutput properties and/or labels such as different organisms or cell lines [25–37]. Sizochenko et al. reported a new methodology for NP safety estimation in different organisms [38]. Predicting NP safety instead of biological activity has been the objective of other studies as well [37,39].

As a new strategy to tackle this problem, González-Díaz et al. have developed IFPTML, a multioutput, and input-coded multilabel ML method, which stands for information fusion (IF) + perturbation theory (PT) + machine learning (ML) algorithm [40]. In recent investigations, the IFPTML model has shown to be a powerful tool in molecular sciences and NDD research for the analysis of big datasets that include both structural and non-structural parameters. Application examples are drug screening, protein targeting, the prediction of coated-NP drug release systems [41–49], multitarget networks of neuroprotective compounds for a theoretical study of new asymmetric 1,2-rasagiline carbamates [50], a TOPS-MODE model of multiplexing neuroprotective effects of drugs, an experimental/theoretical study of new 1,3-rasagiline derivatives potentially useful in neurodegenerative diseases [51], as well as QSAR and complex networks in pharmaceutical design, microbiology, parasitology, toxicology, cancer, and neurosciences [52]. Furthermore, this new model also has been used for very similar systems to this research work such as NP systems, taking into account NP structure and coating agents, synthesis conditions of NPs and loaded drugs, cancer co-therapy drugs, or assay conditions [53–57]. Here we developed IFPTML models for the proposal of N2D3Ss containing NDD and NP components.

## Results and Discussion

**Figure 1:**
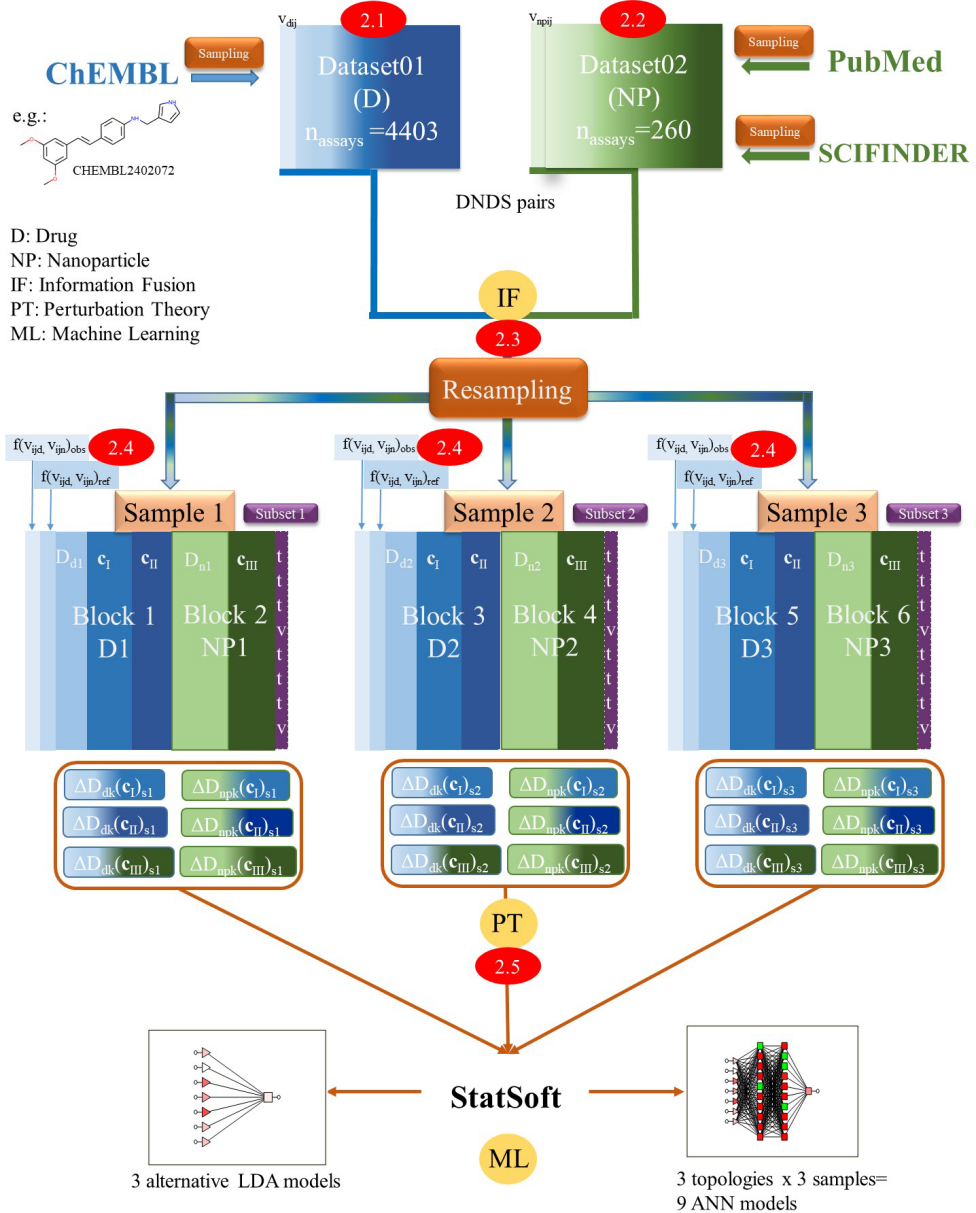
Detailed information processing workflow of the IFPTML models. Steps 2.1 and 2.2: data collection (ChEMBL dataset of NDDs and NP cytotoxicity dataset); step 2.3: data pre-processing and information fusion (NP and NDD assays); step 2.4: definition of objective and reference functions; step 2.5: calculation of the perturbation theory operator (PTO).

In order to build the IFPTML models we carried out the steps shown in Figure 1, which shows the general workflow of all computational procedures in this study. For a better understanding of all steps, we enumerated them with 2.1, 2.2., and so on.

**Figure 2:**
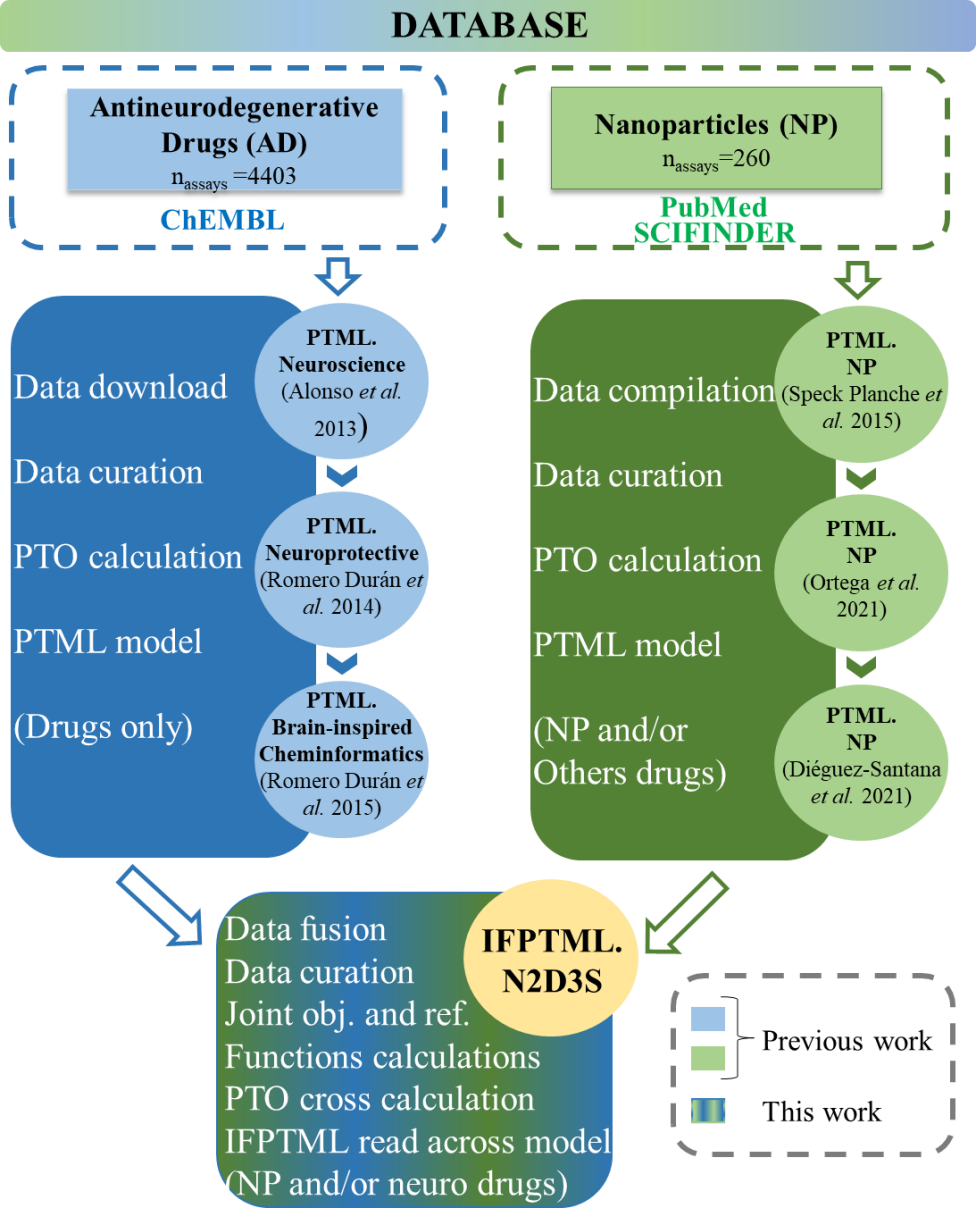
Connection of the current IFPTML model to other PTML models developed by our research group.

Figure 2 shows the connections regarding methodology and used databases to our previous publications. For each PTML model development, data download/compilation, data curation, and so on were carried out separately by researchers. First, the database of antineurodegenerative drugs (ADs) was downloaded from ChEMBL by Alonso and coworkers. These researchers employed this database to create advanced predictive models known as multitarget or multiplexing QSAR. These models are designed to forecast both the potential neurotoxicity and neuroprotective effects of drugs across various experimental setups, including multiple assays, drug targets, and model organisms [41]. Later, Romero Durán et al. enriched the AD database and constructed multitarget networks of neuroprotective compounds to study new asymmetric 1,2-rasagiline carbamates. These authors developed a TOPS-MODE model to analyze the multiple neuroprotective effects of drugs and to conduct experimental/theoretical studies on new 1,3-rasagiline derivatives potentially useful in neurodegenerative diseases [50]. Additionally, Romero Durán et al. expanded the AD database to develop artificial neural network (ANN) algorithms. These models were designed to forecast how ADs interact with targets within the CNS interactome [58]. Speck-Planche et al. compiled manually a database of NPs from the literature. They constructed a QSAR model to investigate multiple antibacterial profiles of NPs under diverse experimental conditions. Furthermore, Ortega-Tenezaca et al. enriched the NP dataset and developed a PTML model for the discovery of antibacterial NPs [59]. Diéguez et al. expanded the NP database and developed a PTML model in order to design antibacterial drug and NP systems [10].

In this study, we utilized the IFPTML model to investigate N2D3Ss, encompassing assays of ADs and preclinical assays for NPs. To achieve this, we conducted the IF of AD and NP databases, curated the data, combined the objective and reference functions, and calculated the PTO.

### NDDs ChEMBL dataset

First, we collected the data of preclinical assays for NDDs from the ChEMBL dataset (see step 2.1. in Figure 1) [60–62]. This dataset contained 4403 preclinical assays for 2566 NDDs (unique drugs), that is, approximately 1.71 assays for each drug. The information downloaded from ChEMBL included discrete variables *c*_d_*_j_* used to specify the conditions/labels of each assay. These variables are *c*_d0_, the biological activity parameter, *c*_d1_, the target protein involved in NDs, *c*_d2_, the cell line for NDD assays, and *c*_d3_, the model organism. Each one of these assays included one out of *n*(*c*_d0_) = 46 possible biological activity parameters (e.g., EC_50_ or *K*_i_ (nM)). They also involved some of the *n*(*c*_d1_) = 21 target proteins, *n*(*c*_d2_) = 7 cell lines (SH-SY5Y, CHO-K1, HEK293, PC-12, CHO, HEK-293T, and HuT78), and *n*(*c*_d3_) = 7 model organisms (*Homo sapiens, Rattus norvegicus, Mus musculus, Cavia porcellus, Canis lupus familiaris, Macacafas cicularis*, and *Caenorhabditis elegans*). The information downloaded from ChEMBL also included another set of discrete variables used to codify the nature/quality of data. These variables are *c*_d4_, the type of target, *c*_d5_, the type of assay, *c*_d6_, the data curation, c_d7_, the confidence score, and c_d8_, the target mapping. Specifically, the target types are *n*(*c*_d4_) = 6 (single protein, organism, tissue, non-molecular target, and ADMET), and the assay types are *n*(*c*_d5_) = 3 (binding, functional, and ADMET). In addition, data curation has *n*(*c*_d6_) = 3 different values (auto-curation, expert, and intermediate), the confidence scores are *n*(*c*_d7_) = 4 (9: direct single protein target assigned, 1: target assigned is non-molecular, 0: default value, that is, target assignment has yet to be curated, and 8: homologous single protein target assigned) and the target mapping is *n*(*c*_d8_) = 3 (protein, non-molecular target, and homologous protein). Furthermore, this database included the molecular descriptor **D**_dk_ = [*D*_d1_, *D*_d2_, *D*_d3_] in order to define the chemical structure of the NDD compound. Specifically, we used two types of molecular descriptor for the *i*-th compound, namely *D*_d1_ = logarithm of the *n*-octanol/water partition coefficient (LOGP*_i_*) and *D*_d2_ = topological polar surface area (PSA*_i_*). The detailed information of this dataset is given in Supporting Information File 1 (datasheet “ChEMBL”).

### NP cytotoxicity dataset

Simultaneously, we downloaded the data of preclinical assays for the cytotoxicity of NPs from different sources (see step 2.2. in Figure 1). We selected 62 papers from the scientific literature databases Pubmed and SciFinder [63–65]. This dataset included 260 preclinical assays for 31 unique NPs. Therefore, the number of assays for each NP is about 8.39. Moreover, the data covered a huge range of properties of NPs such as morphology, physicochemical properties, coating agents, length, and time of assay. These properties were defined as discrete variables *c*_n_*_j_* applied to identify the conditions/labels of each assay. Then, we enumerated all particular conditions of each assay as a general vector **c**_nj_ = [*c*_n1_, *c*_n2_, *c*_n3_,…, *c*_nmax_]. These variables are *c*_n0_, the biological activity parameter, *c*_n1_, the cell line, *c*_n2_, the NP shape, *c*_n3_, the measurement conditions, and *c*_n4_, the coating agent. Each of these assays involved at last one out of *n*(*c*_n0_) = 5 possible biological activity parameters (CC_50_, EC_50_, IC_50_, LC_50_, and TC_50_). They also include *n*(*c*_n1_) = 53 cell lines (e.g., A549 (H), RAW 264.7, and Neuro-2A (M)) and *n*(*c*_n2_) = 10 NP shapes (spherical, irregular, slice-shaped, needles, rods, elliptical, pseudo-spherical, polyhedral, pyramidal, and strips). In addition, they contain *n*(*c*_n3_) = 8 NP measurement conditions (dry, H_2_O, DMEM, RPMI, 1% Trion X-100/H_2_O, H_2_O/TMAOH, egg/H_2_O, and H_2_O/HMT) and *n*(*c*_n4_) = 16 coating agents (UC, PEG-Si(OMe)_3_, PVA, sodium citrate, 11-mercaptoundecanoic acid, PVP, propylamonium fragment, undecylazide fragment, CTAB, *N,N,N*-trimethyl-3(1-propene) ammonium fragment, potato starch, *N*-acetylcysteine, CMC-90, 2,3-dimercaptopropanesulfonate, 3-mercaptopropanesulfonate, and thioglycolic acid). The full information of this dataset is shown in Supporting Information File 1 (datasheet “NP”).

### DNDS pair resampling

#### IF processing of biological parameters

First, we described and acquired the objective value in order to design the IFPTML model for N2D3S. We defined the target function by applying the vectors of descriptors for all cases **D***_k_* to use as the input variable in the ML model. The target function is commonly achieved by a mathematical conversion of the original theoretical or observed feature of the scheme under analysis [66–68]. In this IFPTML model, it includes two groups of observed values, specifically *v*_i_*_j_*(*c*_d0_) and *v*_n_*_j_*(*c*_n0_). In addition, it contains two types of input vectors, **D**_d_*_k_* and **D**_n_*_k_*, for the preclinical NDD and NP assays, respectively. Moreover, in this dataset was a large number of different biological parameters *c*_d0_ and *c*_n0_. For example, there are properties such as half the maximum inhibitory concentration (IC_50_ (nM)), half the maximum effective concentration (EC_50_ (nM)), or the lethal concentration of a substance for an organism (LC_50_ (nM)). Another difficulty is that the majority of *v*_i_*_j_*(*c*_d0_) and *v*_n_*_j_*(*c*_n0_) values collected are numbers with decimals. Furthermore, in order to acquire the optimum N2D3S, we prioritize some properties and deprioritize others. In this context, we introduced a “desirability” parameter to tackle this problem

The desirability value was established as *d*(*c*_d0_) = 1 or *d*(*c*_n0_) = 1 when the value of *v*_i_*_j_*(*c*_d0_) or *v*_n_*_j_*(*c*_n0_) needs to be maximized, otherwise *d*(*c*_d0_) = −1 or *d*(*c*_n0_) = −1. The different NDD and NP properties/characteristics possess a large number of designations or labels *c*_d0_ and *c*_n0_, respectively, and increase the unreability of the data, making it more laborious to build a regression model. For example, in context of a specific case, biological activity parameters *c*_d0_ with *d*(*c*_d0_) = 1 are Bmax (fmol/mg), the total number of receptors expressed in the same units, activity (%), and Cp (nM). Whereas parameters with *d*(*c*_d0_) = −1 are, for example, EC_50_ (nM), IC_50_ (nM), and Imax (%). To address this problem, we used a cutoff value to divide AD and NP assays into favorable and non-favorable assays. It is worth mentioning that using a cutoff is a common practice in drug discovery processes. As a result, acquiring the final target function, the pre-processing of all observed *v*_i_*_j_*(*c*_d0_) and *v*_n_*_j_*(*c*_n0_) values is crucial in order to remove or reduce imprecisions. Eventually, IF processing of the parameters *v*_i_*_j_*(*c*_d0_) and *v*_n_*_j_*(*c*_n0_) enabled us to obtain a target function of the N2D3Ss.

We also used a cutoff to rescale the parameters of *v*_i_*_j_*(*c*_d0_) and *v*_n_*_j_*(*c*_n0_) to obtain the Boolean (dummy) functions *f*(*v*_i_*_j_*(*c*_d0_))_obs_ and *f*(*v*_n_*_j_*(*c*_n0_))_obs_. These values were obtained as *f*(*v*_i_*_j_*(*c*_d0_))_obs_ = 1 if *v*_i_*_j_*(*c*_d0_) > cutoff and *d*(*c*_d0_) = 1, or *v*_i_*_j_*(*c*_d0_) < cutoff and desirability *d*(*c*_d0_) = −1; otherwise *f*(*v*_i_*_j_*(*c*_d0_)) = 0. Similarly, *f*(*v*_n_*_j_*(*c*_n0_))_obs_ = 1 if *v*_n_*_j_*(*c*_n0_) > cutoff and *d*(*c*_n0_) = 1, or *v*_n_*_j_*(*c*_n0_) < cutoff and *d*(*c*_n0_) = −1; else *f*(*v*_i_*_j_*(*c*_d0_), *v*_n_*_j_*(*c*_n0_)) = 0. The values *f*(*v*_i_*_j_*(*c*_d0_))_obs_ = 1 and *f*(*v*_n_*_j_*(*c*_n0_))_obs_ = 1 mean to have a positive desired effect of both NDDs and NPs. As a result, the target function was described as *f*(*v*_i_*_j_*(*c*_d0_), *v*_n_*_j_*(*c*_n0_))_obs_ = *f*(*v*_i_*_j_*(*c*_d0_))_obs_·*f*(*v*_n_*_j_*(*c*_n0_))_obs_. Therefore, the outcome of the IF scaling *f*(*v*_i_*_j_*(*c*_d0_), *v*_n_*_j_*(*c*_n0_))_obs_ is determined by the *i*-th NDD compound and the *n*-th NP measurement conditions. The remaining cases, *f*(*v*_i_*_j_*(*c*_d0_), *v*_n_*_j_*(*c*_n0_))_obs_ = 0, indicate that at least one of the abovementioned conditions fail.

### Definition of objective and reference functions

#### IF phase for combining the references

**Figure 3:**
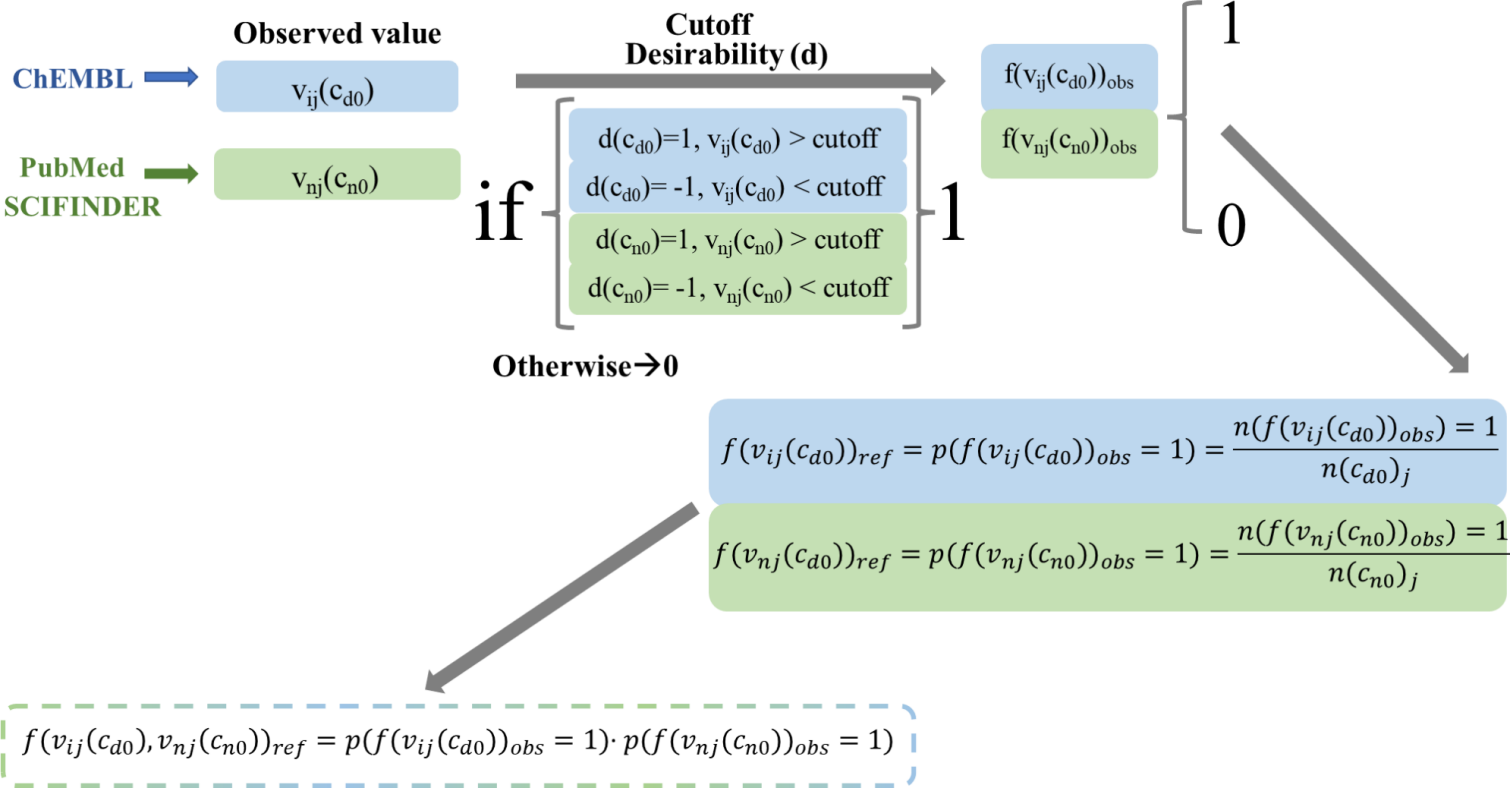
Reference function calculation workflow.

After we obtained the target function, the next step is to describe the input variables of the IFPTML model. Input variable for this model is the reference function *f*(*v*_i_*_j_*(*c*_d0_), *v*_n_*_j_*(*c*_n0_))_ref_. The function *f*(*v*_i_*_j_*(*c*_d0_), *v*_n_*_j_*(*c*_n0_))_ref_ plays an important role because this function characterizes the expected probability *f*(*v*_i_*_j_*(*c*_d0_), *v*_n_*_j_*(*c*_n0_))_ref_ = *p*(*f*(*v*_i_*_j_*(*c*_d0_), *v*_n_*_j_*(*c*_n0_))_ref_ = 1) for achieving the required level of activity for a specific property acquired from well-known systems. IFPTML uses values from well-known systems or subset systems as reference. Afterwards, this model includes the effect of different deviations (perturbations) of the query function from the reference function. Accordingly, *f*(*v*_i_*_j_*(*c*_d0_), *v*_n_*_j_*(*c*_n0_))_ref_ can be considered a function related to observed (not predicted) outcomes. In the above section, we mentioned the step of IF scaling to transform the original *v*_i_*_j_*(*c*_d0_) and *v*_n_*_j_*(*c*_n0_) values into *f*(*v*_i_*_j_*(*c*_d0_))_obs_ and *f*(*v*_n_*_j_*(*c*_n0_))_obs_ functions. When we acquire *f*(*v*_i_*_j_*(*c*_d0_))_obs_ and *f*(*v*_n_*_j_*(*c*_n0_))_obs_ for all cases in our dataset, the next step is to quantify each of the positive outcomes *n*(*f*(*v*_i_*_j_*(*c*_d0_))_obs_ = 1) and *n*(*f*(*v*_n_*_j_*(*c*_n0_))_obs_ = 1). Subsequently, in order to obtain the reference or expected functions (Figure 3), we divide the previous values by the entire number of cases for the NDD and NP systems separately. We describe these functions as *f*(*v*_i_*_j_*(*c*_d0_))_ref_ = *p*(*f*(*v*_i_*_j_*(*c*_d0_))_obs_ = 1) = *n*(*f*(*v*_i_*_j_*(*c*_d0_))_obs_ = 1)/*n*(c_d0_)*_j_* and *f*(*v*_n_*_j_*(*c*_n0_))_ref_ = *p*(*f*(*v*_n_*_j_*(*c*_n0_))_obs_ = 1) = *n*(*f*(*v*_n_*_j_*(*c*_n0_))_obs_ = 1)/*n*(c_n0_)*_j_*. In this context, we can calculate the reference function directly to recognize the probability products for both subsystems *f*(*v*_i_*_j_*(*c*_d0_), *v*_n_*_j_*(*c*_n0_))_ref_ = *p*(*f*(*v*_i_*_j_*(*c*_d0_), *v*_n_*_j_*(*c*_n0_))_obs_ = 1) = *p*(*f*(*v*_i_*_j_*(*c*_d0_))_obs_ = 1)·*p*(*f*(*v*_n_*_j_*(*c*_n0_))_obs_ = 1). It is worth mentioning that the usage of the reference function at this point is another representation of the IF (combination) of NDD and NP datasets.

### PTO calculation

#### IFPTML N2D3S data analysis

As we mentioned in the previous section, we acquired the results of many cytotoxicity preclinical assays of different NPs [69–70]. Complementarily, we obtained the data of preclinical assays for NDDs from the ChEMBL database [60,71–72]. It included the calculation of the vectors **D**_n_*_k_* and **D**_d_*_k_* of structural descriptors for all NPs and NDDs. In addition, we constructed the vectors **c**_n_*_j_* and **c**_d_*_j_* in order to list each label and assay condition for all preclinical assays of NPs and NDDs. Subsequently, we obtained the values Δ*D*_d_*_k_*(**c**_d_*_j_*) and Δ*D*_n_*_k_*(**c**_n_*_j_*) of the respective moving average deviation PTOs.

The NDD vector lists each element ***D***_d_*_k_* = [*D*_d1_, *D*_d2_]. Precisely, these elements are the NDD structural descriptors, which have enabled the development of various strategies to characterize and classify the structure of potential bioactive molecules [73]. These structural descriptors are *D*_d1_ = logarithm of the *n*-octanol/water partition coefficient (LOGP_i_) and *D*_d2_ = topological polar surface area (PSA*_i_*). In contrast, the cytotoxicity NP vector lists the elements as **D**_n_*_k_* = [*D*_n1_, *D*_n2_, *D*_n3_, *D*_n4_, *D*_n5_, *D*_n6_, *D*_n7_, *D*_n8_, *D*_n9_, *D*_n10_, *D*_n11_, *D*_n12_, *D*_n13_, *D*_n14_, *D*_n15_, *D*_n16_, *D*_n17_, *D*_n18_, *D*_n19_, *D*_n20_]. Specifically, they are *D*_n1_ = NMUn (number of monomer units), *D*_n2_ = Lnp (NP length), *D*_n3_ = Vnu (NP volume), *D*_n4_ = Enu (NP electronegativity), *D*_n5_ = Pnu (NP polarizability), *D*_n6_ = Uccoat (unsaturation count), *D*_n7_ = Uicoat (unsaturation index), *D*_n8_ = Hycoat (hydrophilic factor), *D*_n9_ = AMR coat (Ghose–Crippen molar refractivity), *D*_n10_ = TPSA(NO)coat (topological polar surface area using N,O polar contributions), *D*_n11_ = TPSA(Tot)coat (topological polar surface area using N,O,S,P polar contributions), *D*_n12_ = ALOGPcoat (Ghose–Crippen octanol/water partition coefficient), *D*_n13_ = ALOGP2coat (squared Ghose–Crippen octanol/water partition coefficient (logP^2)), *D*_n14_ = SAtotcoat (total surface area from P_VSA-like descriptors), *D*_n15_ = SAacccoat (surface area of acceptor atoms from P_VSA-like descriptors), *D*_n16_ = SAdoncoat (surface area of donor atoms from P_VSA-like descriptors), *D*_n17_ = Vxcoat (McGowan volume), *D*_n18_ = VvdwMGcoat (van der Waals volume from McGowan volume), *D*_n19_ = VvdwZAZcoat (van der Waals volume from the Zhao–Abraham–Zissimos equation), and *D*_n20_ = PDIcoat (packing density index).

#### PT data preprocessing

Apart from the vectors **D**_d_*_k_* and **D**_n_*_k_*, the IFPTML study takes into account all vectors **c**_d_*_j_* and **c**_n_*_j_* as parts of the non-numerical experimental conditions and labels for both NDD and NP preclinical assays. We calculated the PTOs of the NDD and NP preclinical assays including this additional information. We used Equation 1 and Equation 2 in order to obtain the moving average (MA) PTOs of NDDs and NPs. The PT model begins with the expected value of a well-known activity and adds the effect of different perturbations/variations to the system. Consequently, the model includes two different input variables, namely the reference or expected-value function *f*(*v_ij_*)_ref_ and the PT operators Δ*D_k_*(*c_j_*). Specifically, they are applied for accounting structural and assay information on NDDs and NPs. In addition, the PTOs Δ*D*(D_d_*_k_*) and Δ*D*(D_n_*_k_*) label structural and/or physicochemical characteristics of NDDs and NPs on the variables Δ*D*(*D*_d_*_k_*) and Δ*D*(*D*_n_*_k_*), respectively. Furthermore, the PTOs Δ*D*(*D*_d_*_k_*) and Δ*D*(*D*_n_*_k_*) classify biological assay data of NDDs and NPs with the variables ⟨*D*(*D*_d_*_k_*)**_c_**_d_*_j_*⟩ and ⟨*D*(*D*_n_*_k_*)**_c_**_n_*_j_*⟩, respectively. ⟨*D*(*D*_d_*_k_*)⟩ and ⟨*D*(*D*_n_*_k_*)⟩ are the representations of the average operator for counting all cases with the equivalent subset of methodology conditions **c**_d_*_j_* and **c**_n_*_j_*, respectively. Accordingly, they ought to provide exact values for a particular assay with minimum one altered element in methodology conditions of the vectors **c**_d_*_j_* or **c**_n_*_j_*. In this regard, they can specify which assay we are referring to [53–57]. Another kind of PTOs involved in this model is the NDD–NP coating agent moving average balance (MAB) PTO ΔΔ*D*(*D*_ca1_, *D*_ca2_, *D*_d_*_k_*) (Equation 3). The MAB PTO takes into consideration the likenesses between the information on NDDs and the NP coating agent. Furthermore, PTOs centered straightly on MA and/or linear and non-linear conversions of MA have been applied for NDD and NP development in previous research work [49,55–56]. The MAS is another way of expressing the combination of IF and PT cumulative procedures of NDD and NP datasets.


[1] $$\Delta D\left(D_{\text{d}k}\right)=D\left(D_{\text{d}k}\right)-\langle D{\left(D_{\text{d}k}\right)}_{c_{\text{d}j}}\rangle$$

[2] $$\Delta D\left(D_{\text{n}k}\right)=D\left(D_{\text{n}k}\right)-\langle D{\left(D_{\text{n}k}\right)}_{c_{\text{n}j}}\rangle$$

[3]

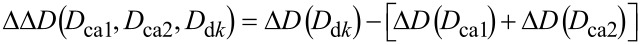



#### IF phase and proposal of training and validation series subsets

To develop the ML models, each of the sample cases are assigned to either the training (subset t) or validation (subset v) series. The process of assignment ought to be random, illustrative, and stratified [74]. Because of the nature of this combinatory system, our sampling also has to take into account the IF scaling procedure. Initially, we obtained the NDD activity dataset from the open database ChEMBL, which has been compiled from primary published literature. The preclinical NP cytotoxicity assays were acquired from journal articles. Afterwards, we prepared each case as the following labels c_d0_, c_d1_, c_d2_, c_d3_, c_d4_, c_d5_, c_d6_, c_d7_, c_d8_, c_n0_, c_n1_, c_n2_, c_n3_, and c_n4_. These cases were organized by ranking the labels alphabetically from A to Z (as we mentioned before, they are non-numeric variables in nature). The preference order of the labels on the procedure of ranking was c_d0_ → c_n0_ → c_d1_ → c_n1_ → c_d2_ → c_n2_→ c_d3_ → c_n3_. In other words, we organized the cases first by c_d0_, then by c_n0_, and so forth. This preference order considers the IF step by interchanging labels from AD and NP datasets. Afterwards, we assigned three quarters of the cases to subset t and the remaining quarter to subset v. This random assignment improves the likelihood that nearly all categories of individual labels are denoted by subsets t and v (stratified or proportional random sampling). In addition, this boosts the possibility that practically all cases for each label are in a distribution of 3/4 in subset t and 1/4 subset v, known as representative sampling. It is worth mentioning that the 75% and 25% proportion between training and validation is the most used one in big data analysis [74].

#### IFPTML-LDA model

The IFPTML N2D3S model utilizes as input variables the PTOs specified in the previous section to codify information of the putative N2D3Ss with their corresponding subsystems NDD and NPs. Combining objective function *f*(*v*_i_*_j_*, *v*_n_*_j_*)_obs_ and reference function *f*(*v*_i_*_j_*, *v*_n_*_j_*)_ref_ and adding the IF PTOs ΔΔ*D*(*D*_c1_, *D*_c2_, *D*_d_*_k_*), we obtained the output function *f*(*v*_i_*_j_*, *v*_n_*_j_*)_calc_. This function carries out dataset crosscut classification of NDD and NP information. The generic equation for the IFPTML linear model is the following (Equation 4):


[4] $$\begin{array}{c} f{\left(v_{\text{i}j},v_{\text{n}j}\right)}_{\text{calc}}=a_{0}+a_{1}\cdot f{\left(v_{\text{i}j},v_{\text{n}j}\right)}_{\text{ref}}\\ +\sum_{k=1,j=1}^{k=k_{\max},j=j_{\max}}a_{k,j}\cdot \Delta D{\left(D_{k\text{i}}\right)}_{c_{\text{d}j}}\\ +\sum_{k=1,j=1}^{k=k_{\max},j=j_{\max}}a_{k,j}\cdot \Delta D{\left(D_{k\text{n}}\right)}_{c_{\text{n}j}}\\ +\sum_{k=1,j=1}^{k=k_{\max},j=j_{\max}}a_{k,j}\cdot \Delta \Delta D{\left(D_{k\text{i}},D_{k\text{n}}\right)}_{c_{\text{d}j},c_{\text{n}j}} \end{array}$$

#### Generalities for IFPTML model training and validation series

In many big data systems, the linear discriminant analysis (LDA) model is the most commonly used tool to seek the preliminary model because of the simplicity of this technique. In this regard, within this model we applied a forward stepwise (FSW) [75] process that can select automatically the most essential input variables for N2D3Ss. We obtained all results by using the software STATISTICA 6.0 [74]. Afterwards, we applied the expert-guided selection (EGS) heuristic [76] in order to retrain the LDA method using the most crucial parameters selected by the FSW process along with other missing aspects. All IFPTML models were obtained by calculating different statistical parameters, specifically sensitivity (Sn), specificity (Sp), accuracy (Ac), chi-square (*χ^2^*), and the *p*-level [77–78].

#### IFPTML-LDA vs cross linear model

In the Introduction section, we indicated the use of ML approaches as a promising strategy in order to tackle practical problems of nanotechnology, such as reducing the number of experiments [79–84]. In this paper the IFPTML method was used to combine preclinical assays of NDDs and NPs*.* Speck-Planche et al. described multiple IFPTML approaches regarding toxicity and drug delivery of NPs with a large number of species under a wide variety of experimental conditions. However, this study did not take into account the NDDs [54,69,85]. In contrast, Nocedo-Mena et al. reviewed an IFPTML method to explore the activity of NDDs against numerous species and under different assay conditions; but this research they did not consider NPs as part of the system [86]. Accordingly, these models could not take into consideration both components (NDD and NPs) of the N2D3Ss. In our group, Dieguéz-Santana et al. for the first time applied successfully the IFPTML technique to study the combination of multiple antibacterial drugs and preclinical assays on the cytotoxicity of NPs [10]. In this paper, we used this new approach to develop complex N2D3Ss containing NDDs and NPs, taking into account, among other things, NDD assays, NP types including coating agents, and NP morphologies. To complete the IF scaling process, we calculated the objective function *f*(*v*_i_*_j_*, *v*_n_*_j_*)_obs_ = *f*(v_i_*_j_*)_obs_·*f*(v_n_*_j_*)_obs_. The main purpose of this function is to increase the effect of certainty and maintain the homogeneity of scales. Once the PTOs were obtained, we applied ML methods so as to fit *f*(*v*_i_*_j_*, *v*_n_*_j_*)_obs_ and to achieve the IFPTML models. As indicated in the previous section, we classified the preclinical NDD assays, **c**_dj_, onto two different partitions (subsets) of variables **c**_I_ and **c**_II_. The partition **c**_I_ defines the biological characteristics; it contains, among other things, *c*_d0_ = biological activity parameters of NDDs (e.g., IC_50_, *K_i_*, potency, and time) and *c*_d1_ = type of proteins involved in the NDs*.* The partition **c**_II_ defines the data quality; it contains, among other things, c_d4_ = type of target and c_d5_ = type of assay*.* For the preclinical NP cytotoxicity assays, **c**_n_*_j_* forms only one partition **c**_III_, which describes its nature and involves c_n0_ = biological activity parameters of the NPs (e.g., CC_50_, IC_50_, LC_50_, and EC_50_), c_n1_ = cell lines, c_n2_ = NP morphology, and c_n3_ = NP synthesis conditions. In addition, we acquired two types of IFPTML-LDA model for designing the N2D3Ss. On the one hand, we obtained the IFPTML-LDA by calculating the PTOs Δ*D_k_*(**c***_j_*) as the difference between the average value ⟨*D_k_*(**c***_j_*)⟩ and the partition **c**_n_ within of their own set. As result, the best IFPTML-LDA model found is as follows (Equation 5):


[5]

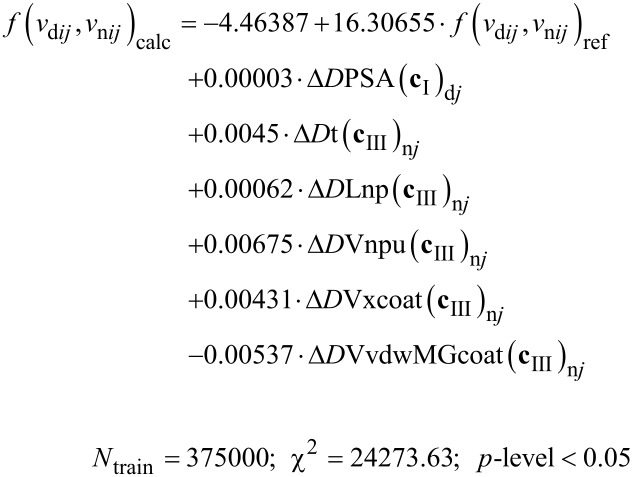



On the other hand, we tested the possibility to improve the results of statistical parameters for the IFPTML-LDA algorithm. To this end, we calculated the PTOs Δ*D_k_*(**c***_j_*) by performing all possible combinations among the average values ⟨*D_k_*(**c***_j_*)⟩ of both vectors **D**_n_*_k_* and **D**_d_*_k_* with each partition. As a result, we obtained three different combinations of crossing PTOs for each sample, one for NDDs (Δ*D*_d_*_k_*(**c**_III_)) and two for NPs (Δ*D*_n_*_k_*(**c**_I_) and *ΔD*_n_*_k_*(**c**_II_)). For simplicity, they are named “IFPTML-LDA with cross” (see more details in Figure 1). The best IFPTML-LDA found with the cross model is the following (Equation 6):


[6]

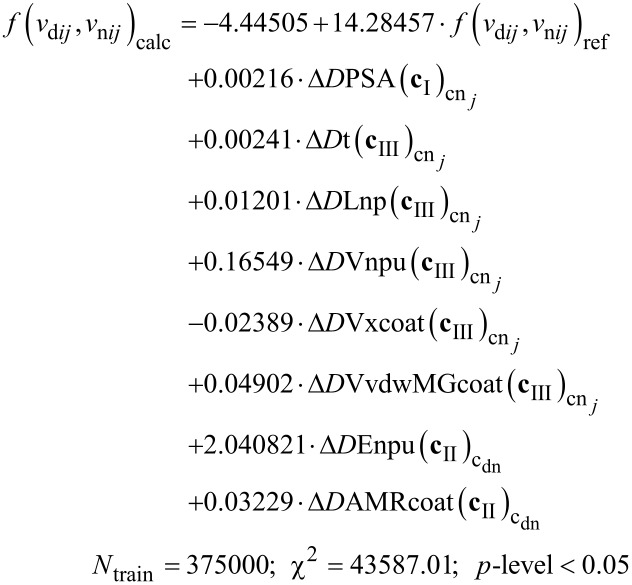



The output function *f*(*v*_d_*_ij_*, *v*_n_*_ij_*)_calc_ provides a real numeric value that will probably be applied to counting N2D3Ss. This function was acquired by calculating the objective function *f*(*v*_i_*_j_*(*c*_d0_), *v*_n_*_j_*(*c*_n0_))_obs_ with the ML method making use of the PTOs. The characteristic of the IFPTML models was defined by the statistical parameters sensibility (Sn), specificity (Sp), accuracy (Ac), chi-square test (χ*^2^*), and *p*-level [74]. The results summary collected in Table 1 contains the statistical parameters for the best models found (Equation 2) for each sample (standard IFPTML-LDA and IFPTML-LDA with cross) are collected in Table 1. The statistical parameters obtained for both methods were in the accuracy range described for the classification model of ML algorithms [77–78]. The standard IFPTML-LDA contains all indispensable variables for defining the NDD structures and the most significant parameters for NPs, such as morphology, size, and assay conditions, among other things. In the IFPTML-LDA with cross system, we included not only all essential variables but also two crossing PTOs. These new PTOs were chosen by the FSW method, which can select the most influential variable in the system under study.

**Table 1:** IFPTML-LDA N2D3S model results summary.

Data				Stat.	Param.	Without cross Subset predicted		Param.	With cross Subset predicted	

Sample	Set	Subset	Param.		(%)	0	1	(%)	0	1

1	t	0	Sp		73	255190	94292	72.2	252534	97042
		1	Sn		71	7398	18120	74.4	6517	18907
	v	0	Sp		73.3	85369	31125	72.3	84183	32315
		1	Sn		70.3	2522	5984	73.9	2218	6284

2	t	0	Sp		70	244548	105076	79.5	277907	71717
		1	Sn		62.1	9528	15848	70.1	7584	17792
	v	0	Sp		70	81640	35009	79.7	92929	23720
		1	Sn		63.1	3081	5270	70.7	2451	5900

3	t	0	Sp		70.6	246551	102809	79.6	277921	71439
		1	Sn		62.3	11616	15974	70.1	7668	17972
	v	0	Sp		70.7	82370	34174	79.6	92726	23818
		1	Sn		62.7	3828	5300	70.4	2500	5956

Avg.	t	0	Sp		71.2	248763	100726	77.1	269454	80066
		1	Sn		65.1	9514	16647	71.5	7256	18224
	v	0	Sp		71.3	83126	33436	77.2	89946	26618
		1	Sn		65.4	3144	5518	71.7	2390	6047

The IFPTML-LDA model in this paper had Sn and Sp values of 70%–73% in both training and validation series. The IFPTML-LDA with cross model showed significantly higher Sn and Sp values of 70%–80% in both series. By only adding two PTOs to the standard model, the IFPTML-LDA Sp value was improved by almost 7% in the training/validation series. However, the Sp and Sn values of the “with cross” model are slightly unbalanced in comparison with the standard model; yet, the Sp and Sn values remain approximately constant within the same training and validation series.

#### Linear vs non-linear IFPTML models

In order to obtain the artificial neural network (ANN) model, we used the same PTO variables as in the LDA model. As an alternative to the non-linear models, we created the ANN by using the same software STATISTICA. The ANN can also be used as a new strategy to confirm and validate the linear hypothesis. Both are comparable because the linear neural network (LNN) techniques are analogous to LDA models and they are linear equations. Accordingly, the IFPTML-LNN model is a useful tool to assess the degree of strength of the linear relationship between PTOs and the N2D3S objective function. The IFPTML-LNN models in this work showed lower Sn and Sp values of 64%–65% in the training and validation series, compared with the IFPTML-LDA models, see details in Table 2.

**Table 2:** The best result of IFPTML-ANN N2D3Ss models found.

Sample	IFPTML-ANN Models^a^	Subset	Stat.	Val. (%)	*f*(*v*_i_*_j_*(*c*_d0_), *v*_n_*_j_*(*c*_n0_)) Pred.	Observed		AUROC
						1	0	

01	LDA 7:7-1:1 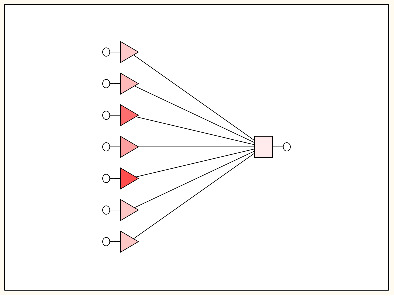 FSTW + EGS	t	Sp	0	73.0	94272	255178	—
			Sn	1	71.0	18057	7367	
		v	Sp	0	73.3	31125	85319	—
			Sn	1	70.3	5980	2522	
	
	MLP 7:7-11-1:1 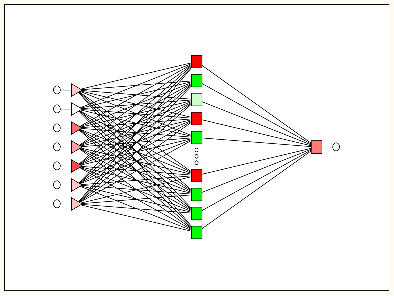 BP96b	t	Sp	0	86.1	300836	48740	0.943
			Sn	1	85.8	3610	2181	
		v	Sp	0	86.1	100278	16220	0.934
			Sn	1	86.2	1173	7329	
	
	DLN 7:7-10-10-1:1 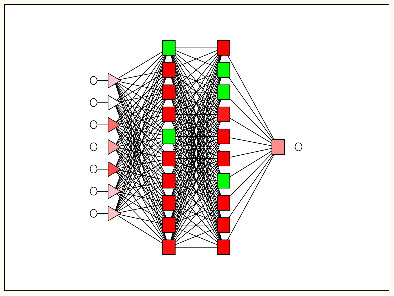 BP100,CG20b	t	Sp	0	85.8	299942	49634	0.945
			Sn	1	85.8	3621	21803	
		v	Sp	0	85.9	100103	16395	0.933
			Sn	1	86.3	1168	7334	
	
	LNN 7:7-1:1 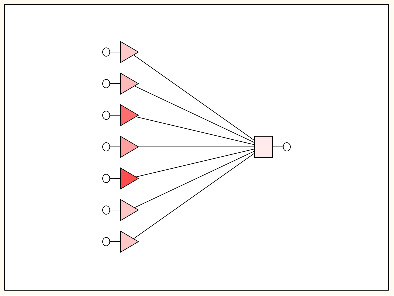 PI	t	Sp	0	65.0	227184	122392	0.744
			Sn	1	64.7	8971	16453	
		v	Sp	0	65.1	75788	40710	0.733
			Sn	1	64.1	3055	5447	

**Figure 4:**
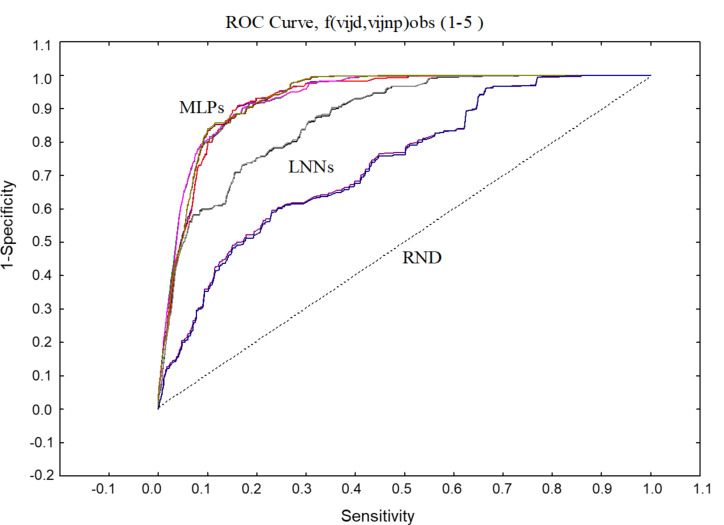
AUROC exploration of IFPTML-MLP and IFPTML-LNN models.

Analogous to the IFPTML-LDA model, the values of the statistical parameters Sp and Sn are considerably balanced and stay steady when comparing training and validation series. Also, we obtained two types of non-linear models, the multilayer perceptron (MLP) and the depth learning network (DLN). The MLP is made up by seven PTOs as input layer, a hidden layer with eleven neurons, and an output layer. The most notable difference is that the DLN involves two hidden layers, each one with ten neurons. Both MLP and DLN showed high Sp and Sn values of 85%–86% in the training and validation series. If we compare the linear IFPTML-ANN model with non-linear models based on the results of statistical parameters, we can confirm that N2D3S is a non-linear system. Another result obtained in the development of the ANN is the area under receiver operating characteristic (AUROC) (Figure 4) [74]. The AUROC curve values are 0.93–0.94 for both MLP and DLN models in the training and validation series. The AUROC values of the non-linear models are remarkably different from the random (RND) curve with AUROC = 0.5 [74].

#### Robustness analysis of IFPTML models

The design of the N2D3Ss involve the combination of a large amount of data on preclinical assays of NDDs and NPs. Because of the nature of this big data system, we divided the information fusion dataset into three samples. In the previous section, we discussed the best model obtained for IFPTML-LDA, IFPTML-LDA with cross and IFPTML-ANN. In this section, a robustness analysis for the three samples is given (see Table 3). In general, the number of cases (*n*) used in training and validation series for all models presented the lowest standard deviation (SDV), which indicated that most of the data in a sample tend to be clustered near its mean [87]. In contrast, the high value of SDV for the DLN model indicates that the data was distributed over a wide range of values. In addition, all models presented similar SDV values in the same training and validation series. Interestingly, the LDA model showed significantly lower values of SDV for Sp (>1), compared with the SDV for Sn (>4) in the training and validation series. However, the SDV values for the LDA cross model were contrary to those of LDA, with lower SDV values for Sn and higher values for Sp. It is worth mentioning that both MLP 1 and LNN models yielded statistical parameters close to its mean, in other words these models are robust. Furthermore, using the IFPTML-ANN model, we also obtained AUROC values as results. After doing the robustness analysis, we can confirm that all AUROC values for all ANN models are robust. In addition, the AUROC graphic (Figure 4) gives evidence to this because of the similarity of the curve shapes.

**Table 3:** Result summary of N2D3Ss alongside average of three samples and standard deviations.

AVG	Model	t			v			AUROC (t/v)

		Sp	Sn	*n*	Sp	Sn	*n*	

	LDA	71.2	65.1	375000	71.3	65.4	125000	—
	LDA cross	77.1	71.5	375000	77.2	71.7	125000	—
	MPL 1	85.1	85.0	375000	85.1	85.1	125000	0.937/0.925
	DNL	79.2	79.0	375000	79.2	79.3	125000	0.893/0.879
	LNN	65.0	64.9	375000	65.1	64.9	125000	0.748/0.737

SDV	Model	t			v			AUROC (t/v)

		Sp	Sn	*n*	Sp	Sn	*n*	

	LDA	1.587	5.082	0	1.739	4.277	0	—
	LDA cross	4.244	2.483	0	4.244	1.940	0	—
	MLP 1	1.266	1.217	0	1.940	1.102	0	0.010/0.010
	DLN	8.489	8.568	0	8.584	8.727	0	0.069/0.071
	LNN	0.100	0.153	0	0.153	0	0	0.005/0.003

The results reveal the strength of the linear hypothesis. Nevertheless, the statistical parameters of the obtained linear model are not satisfactorily at all. As a result, in the IFPTML-LDA with cross model, we enlarged the number of input variables from seven to nine. Thus, we did not obtain substantial change. Therefore, we tested more complex non-linear models so as to improve the Sp and Sn values. The IFPTML-MLP 7:7-11-1:1 model, containing seven input variables in the input layer and eleven neurons in the hidden layer, yielded the best statistical parameters of Sn and Sp values (Table 3). The IFPTML-DLN model, which involves two hidden layers, yielded similar result as IFPTML-MLP 7:7-11-1:1.

Taking into account all the aforementioned results, we can consider both IFPTML-MLP and IFPTML-DLN as the best models with remarkably higher values of Sp and Sn of 85%–86% and AUROC values of 0.93–0.94. However, the DLN model is more complex and yields only a non-significant improvement of statistical parameters in comparison with the MLP model. Thus, we can confirm that N2D3Ss require the MLP model. This selection is supported by the principle of parsimony, prioritizing the simplest explanations among all possible ones [88]. In Table 4, an input variable sensitivity analysis concerning NDDs, NPs, and the corresponding subsystems are shown for the IFPTML-ANN model. The IFPMTL-LNN model involves almost all significant parameters according to the EGS criteria. The majority of parameters provide a substantial influence on the sensitivity ≥ 1 [74]. In many cases, the value of sensitivity analysis is slightly higher with a sensitivity of 1.00–1.08. Nevertheless, the EGS perspective fails in the selection of Δ*D*PSA(**c**_I_) and Δ*D*t(**c**_III_) variables. In this regard, the IFPTML-ANN model suggests that those variables do not affect any model. In contrast, the IFPTML-LNN yielded the lowest value of sensitivity of 1.00–1.13, which would underline the need for a complex model in N2D3Ss. The DLN model involves the essential variables in accordance with the EGS criteria; however, they have remarkably higher sensitivity values of 0.96–2.03. The MLP yielded the highest values of sensitivity between 1.13 and 2.57.

**Table 4:** IFPTML-ANN model input variable sensitivity analysis for different subsystems with their corresponding variables.

Sub-systems	Variables	LNN		MLP		DLN					

		t	v	t	v	t	v	t	v	t	v

NDD_S_&NP	**f**(c_d0_,c_n0_)_ref_	1.02	1.02	1.32	1.33	1.46	1.45	1.25	1.24	1.38	1.40
NDDs	Δ*D*PSA(**c**_I_)	0	0	0	0	0	0	0	0	0	0
NP	Δ*D*t(**c**_III_)	0	0	0	0	0	0	0	0	0	0
	Δ*D*L_np_(**c**_III_)	1.00	1.00	1.14	1.13	1.08	1.08	1.08	1.08	1.60	1.59
	Δ*D*V_npu_(**c**_III_)	1.00	1.00	2.22	2.22	0.92	0.92	1.06	1.05	1.24	1.25
	Δ*D*V_xcoat_(**c**_III_)	1.00	1.00	1.96	1.98	1.45	1.47	1.45	1.48	1.99	2.03
	Δ*D*V_vdw_MG_coat_(**c**_III_)	1.13	1.13	2.57	2.54	1.44	1.43	1.24	1.24	1.91	1.90

#### IFPTML-LDA for N2D3S simulation

In this section, we employed the IFPTML-LDA technique to calculate the probability values for some selected cases of N2D3Ss. The linear model was chosen for its simplicity and the slight improvement of the non-linear model. The value of probability *p*(N2D3S_in_)**_c_**_d_*_j_*_._**_c_**_n_*_j_* was obtained for N2D3Ss, created by the combination of the *i*-th AD*_i_* and the *n*-th NP*_n_*, which are likely to have a desired level of biological activity under both assay conditions **c**_d_*_j_* and **c**_n_*_j_*. This simulation experiment involved in total *N*_N2D3S_ = 88 systems vs a total of *N*_NDDs_ = 123 drugs. Many of these drugs are NDDs with known anti-neurodegenerative activity, generally for Alzheimer and Parkinson diseases. Some of these NDDs are approved by the Food and Drug Administration, while others have been shown to be active in several assays. In addition, the simulation also contained cytotoxicity assays against multiple cell lines, the type of NPs, their coating, and the time of each assay. In this context, we calculated a total of *N*_tot_ = *N*_NDDs_·*N*_NP_ = 22·218 = 4796 values of probability, which were able to predict successfully putative N2D3Ss.

**Figure 5:**
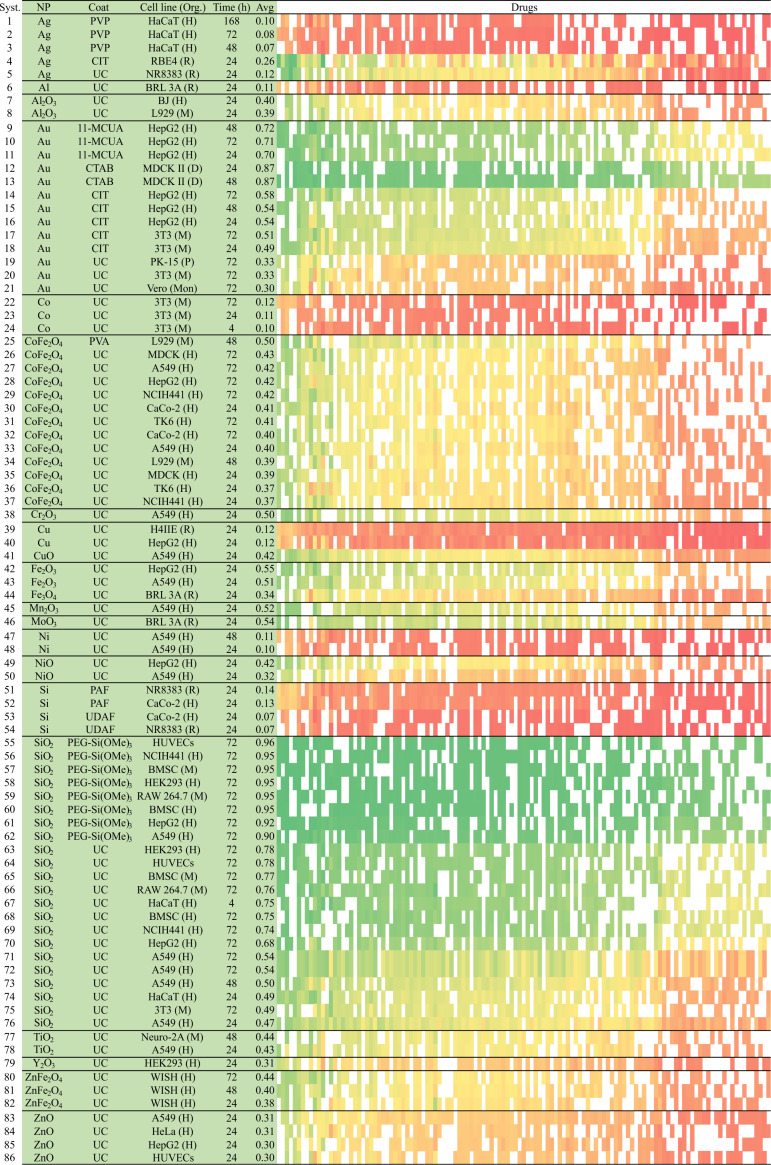
IFPTML-LDA N2D3Ss experiment simulation.

Figure 5 depicts the results in a three-color scale according to the value of probability: the green section indicates high probability (0.61–0.98), yellow low-to-middle probability (0.17–0.60), and red very low probability (<0.17). Assays that have not been reported before, are represented in the original dataset to a very low extent, or whose combination of NDDs and NPs are meaningless were illustrated in white color to avoid an overestimation of results. The results of the IFPTML-LDA model pointed out some N2D3Ss as promising combinations for future additional assays. The resulting N2D3Ss shown in Figure 5 involve twenty different NDDs. The first ten are 1 = clozapine, 2 = galantamine, 3 = levodopa, 4 = apomorphine, 5 = fiduxosin, 6 = beagacestat, 7 = memoquin, 8 = mesodihydroguairetic acid, 9 = tarenflubil, and 10 = huperzine A. The other ten NDDs are 11 = guanidinonaltrindole, 12 = semagacestat, 13 = huprine X, 14= carproctamide, 15= tacrine, 16 = tramiprosate, 17 = preladenant, 18 = piracetam, 19 = istradefylline, and 20 = rivastigmine. These systems include the following coating agents: PEG = polyethylene glycol, PVP = polyvinylpyrrolidone, PPF = propylammonium fragment, and UAF = undecylazide fragment. The symbol UC = uncoated represents non-coated N2D3Ss. Interestingly, a high value of prediction involves PEG-Si(OMe)_3_ as NP coating with *p*(N2D3S_in_)**_c_**_d_*_j_*_._**_c_**_n_*_j_* = 0.80–0.99 for the majority of NDDs. Another important factor that may affect the value of probability is the type of NP. It appears that metal oxide compounds such as SiO_2_ and TiO_2_ along with PEG-Si(OMe)_3_NP coating for almost all NDDs are likely to be promising for further assays. Double metal oxide compounds such as CoFe_2_O_4_ and ZnFe_2_O_4_ obtained intermediate probability values *p*(N2D3S_in_)**_c_**_d_*_j_*_._**_c_**_n_*_j_* = 0.17–0.70 against TK6 (H) and WISH (H). In general, the least advantageous combinations are metal NPs with all NDDs, which give low values of probability (*p*(N2D3S_in_)**_c_**_d_*_j_*_._**_c_**_n_*_j_* = 0.02–0.35). It is worth mentioning that all predictions carried out by this method should be used with caution and require experimental corroboration. The potential utility of the IFPTML method is to speed up experimental studies and to provide inexpensive preliminary results for a large database of N2D3Ss. This approach offers an efficient and powerful tool to direct experimental research as an alternative to tedious trial-and-error tests.

**Figure 6:**
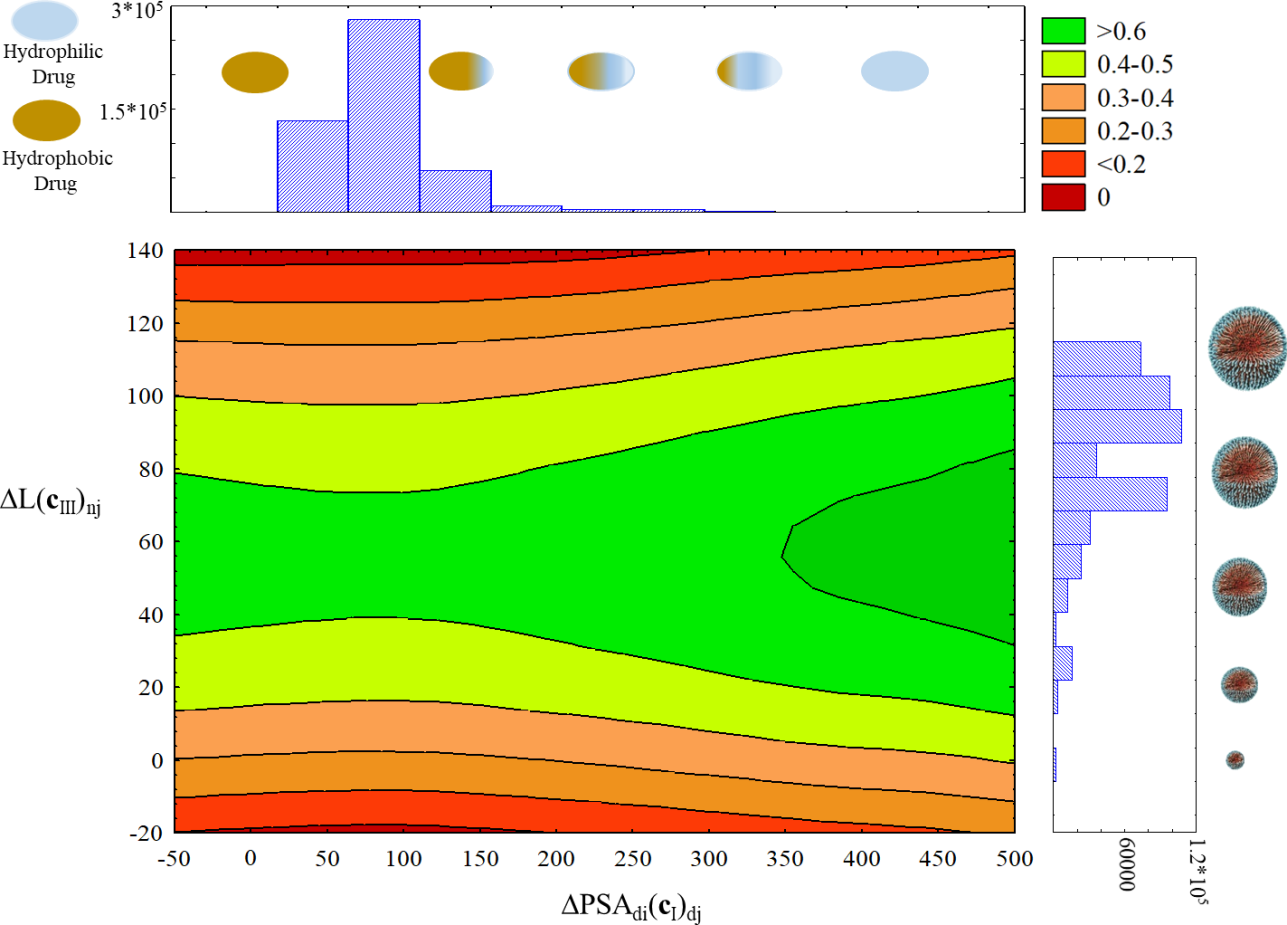
Probability surface scatter plot representing the deviation of NP length considering the partition **c**_III,_, which describes the NP nature and includes c_n0_ = NP biological activity parameters (e.g., CC_50_, IC_50_, LC_50_, and EC_50_), c_n1_ = cell lines, c_n2_ = NP morphology, and c_n3_ = NP synthesis conditions. (Δ*L*(**c**_III_)_n_*_j_*) along with the deviation of NDD hydrophobicity (ΔPSA(**c**_I_)_d_*_j_*) taking into account the partition **c**_I_, which includes the biological characteristics, for example c_d0_ = NDD biological activity parameters (e.g., IC_50_, *K*_i_, potency, and time) and c_d1_ = the type of protein involved in NDs*.*

In addition, the determination of the probability value distribution in a generic sense for the unique pairs of NP cytotoxicity assays and NDDs was carried out. For this, we depict the surface scatterplot of probability values against histograms of NP length along with NDD hydrophobicity (Figure 6). Generally, a third of the probability values remains in the dark green zone, which represents promising N2D3Ss for further assay. It is worth mentioning that most of the cases (white dots) are hydrophobic drugs (on the left of the graph). This feature is one of the most important physicochemical properties for drugs in order to cross the BBB [89]. High lipophilicity can contribute to excessive distribution volumes, increased metabolic liability, and lower unbound drug concentration in the plasma and/or brain; it may also negatively affect pharmaceutical properties, in particular solubility [90]. Most NDDs of this database are in the PSA_d_*_i_* range of 60–120 Å^2^. Stephen et al. suggested that CNS drugs should have a PSA value below 90 Å^2^ for a decent BBB permeability, among other physicochemical characteristics such as number of hydrogen bond donors, molecular size, and shape, with smaller contributions from hydrogen bond acceptors [89]. Although this type of graphic is clearly a simplification of the whole database, it offers simple guidelines for researchers concerned with designing NDD compounds or libraries with improved probability of BBB penetration. The size of the vast majority of NPs for NDD delivery in this database is in the range of 70–115 nm. Recently, Chithrani et al. [91] have demonstrated that size, coating, and surface charge of nanoparticles have a crucial impact on the intracellular uptake process. Similarly, Shilo et al. have investigated the influence of NP size on the probability to cross the BBB by using the endothelial brain cell method. The results indicated that the intracellular uptake of NPs strongly depends on the NP size. This characteristic has a direct impact on biomedical applications. When NPs serve as carriers for drug delivery through encapsulation, a larger NP size (70 nm) is needed. However, when NPs serve as carriers by binding drug molecules to their surface, a larger free surface area is required; therefore, the optimal size would be 20 nm [92]. This principle suggests that a high number of the NPs in our database are proper drug delivery carriers by drug encapsulation.

Thus, the design of new N2D3Ss based on multiple preclinical assays of NP cytotoxicity and NDDs has been carried out successfully. This database involves a high structural and biological diversity, which may help to distinguish active from non-active N2D3Ss. Experimentally, the IFPTML-LDA method predicted with high probability *p*(N2D3S_in_)**_c_**_d_*_j_*_._**_c_**_n_*_j_* > 0.81 all examples reported in Table 5. The results support our initial premise that the IFPTML additive approach is able to carry out an appropriate recognition of N2D3Ss involving additive and synergic cases.

**Table 5:** IFPTML analysis of experimentally tested N2D3S compounds.

Drug^a^	NP	c_d0_ = activity	Δ*D*PSA(**c**_I_)	Obs.^b^	Pred.^c^	*p* ^d^	*L* (nm)^e^

Metal/n.a.							

2234684	Ag	Time (h)	0.57	1	1	0.88	12.50
2376472	Ag	Time (h)	4.30	1	1	0.88	12.50
2234683	Ag	Time (h)	0.57	1	1	0.88	12.50

Metal oxide/n.a.							

3769671	TiO_2_	Cp (nm)	0	1	1	0.94	56
Levodopa	TiO_2_	Time (h)	−3.5	1	1	0.93	56
Sch-58261	TiO_2_	Time (h)	−1	1	1	0.93	56
2180030	TiO_2_	EC_20_ (nm)	0	1	1	0.93	56
Levodopa	TiO_2_	Time (h)	−3.5	1	1	0.93	56
Sch-58261	TiO_2_	Time (h)	−1	1	1	0.93	56
2234689	TiO_2_	Time (h)	0.3	1	1	0.93	56
Morin	TiO_2_	Time (h)	0	1	1	0.93	56

Metal/elliptical							

Datiscetin	Ag	Time (h)	0.3	1	1	0.81	36.8
2234993	Ag	Time (h)	0.4	1	1	0.81	36.8
1240582	Ag	Time (h)	−1.7	1	1	0.81	36.8
1241456	Ag	Time (h)	−2.1	1	1	0.81	36.8

Metal oxide/elliptical							

2180030	Yb_2_O_3_	EC_20_ (nm)	0	1	1	0.90	62.1
Levodopa	Yb_2_O_3_	Time (h)	−3.5	1	1	0.90	62.1
3769671	CeO_2_	Cp (nm)	0	1	1	0.90	44.8

Metal oxide/needle							

3747225	La_2_O_3_	Time (h)	2.8	1	1	0.89	65.8
3769671	La_2_O_3_	Cp (nm)	0	1	1	0.88	65.8

Meta/rod							

3218426	Au	Activity (%)	−2.0	1	1	0.93	37.8
Congo red	Au	Inhibition (%)	3.6	1	1	0.93	37.8
3218189	Au	Activity (%)	−2.0	1	1	0.93	37.8
3580774	Au	Activity (nm)	0	1	1	0.93	37.8

Metal oxide/pyramidal							

PGA^f^	TiO_2_	Time (h)	−18	1	1	0.91	6.5
Apomorphine	TiO_2_	Time (h)	−17	1	1	0.91	50
1801682	TiO_2_	Time (h)	−20	1	1	0.91	50

Metal oxide/irregular							

3350757	TiO_2_	Time (h)	−5.3	1	1	0.93	21
3747225	TiO_2_	Time (h)	2.8	1	1	0.93	21
1243007	TiO_2_	Time (h)	−0.7	1	1	0.92	21
3769671	TiO_2_	Cp (nm)	0	1	1	0.92	21
Levodopa	TiO_2_	Time (h)	−3.5	1	1	0.92	21

Metal Oxide/pseudo-spherical							

2376474	CeO_2_	Time (h)	3.9	1	1	0.89	8
3747225	CeO_2_	Time (h)	2.8	1	1	0.89	8
3769671	CeO_2_	Cp (nm)	0	1	1	0.89	8
Levodopa	CeO_2_	Time (h)	−3.5	1	1	0.89	8
Sch-58261	CeO_2_	Time (h)	−1.0	1	1	0.89	8

Metal/spherical							

2151181	Au	ED_50_ (mg/kg)	−0.4	1	1	0.94	42.9
1222303	Au	ED_50_ (mg/kg)	−0.4	1	1	0.94	42.9
2181911	Au	Activity (%)	1.6	1	1	0.90	42.9
3397881	Au	Inhibition (%)	−1.1	1	1	0.90	42.9
3785241	Au	Inhibition (%)	−1.5	1	1	0.90	42.9
3947919	Au	Activity (%)	1.0	1	1	0.90	42.9
3817925	Au	Inhibition (%)	−0.7	1	1	0.90	42.9
3612821	Au	Inhibition (%)	0.3	1	1	0.90	42.9
2159510	Au	Activity (%)	−0.8	1	1	0.90	42.9
2415095	Au	Inhibition (%)	0.5	1	1	0.90	42.9
436483	Au	Inhibition (%)	1.5	1	1	0.90	42.9
2159511	Au	Activity (%)	−1.2	1	1	0.90	42.9
2349470	Au	Activity (%)	−1.8	1	1	0.90	42.9
3127906	Au	Activity (%)	0.6	1	1	0.90	42.9
Propidium	Au	Inhibition (%)	0.4	1	1	0.90	42.9

Metal oxide/spherical							

3218188	SiO_2_	Activity (%)	91	1	1	0.97	12.5
3087679	SiO_2_	Inhibition (%)	69	1	1	0.97	60
3233831	SiO_2_	Inhibition (%)	58	1	1	0.97	44
510384	SiO_2_	K_i_ (nm)	−30	1	1	0.97	47.5
81999	SiO_2_	K_i_ (nm)	−40	1	1	0.97	36.8
3218425	SiO_2_	Activity (%)	91	1	1	0.97	70
55401	SiO_2_	K_i_ (nm)	−31	1	1	0.97	37
3233829	SiO_2_	Inhibition (%)	58	1	1	0.97	36.8
3087678	SiO_2_	Inhibition (%)	69	1	1	0.97	3.4
3769671	SiO_2_	Cp (nm)	0	1	1	0.99	5.5
2234689	SiO_2_	Time (h)	37	1	1	0.99	36.8
2234690	SiO_2_	Time (h)	37	1	1	0.99	16.4


^a^ChEMBL ID or drug name; the name of the drug is depicted if it is available, otherwise the ChEMLID code of the drug is indicated, which can be easily consulted by accessing the CheMBL website. ^b^Class. Obs: *f*(*v*_i_*_j_*, *v*_n_*_j_*)_obs_. ^c^Class. Pred: *f*(*v*_i_*_j_*, *v*_n_*_j_*)_pred_. ^d^*p*: probability calculated as *p*(N2D3S_in_/**c**_d_*_j_*, **c**_n_*_j_*)_pred_ = 1/(1 + exp[−*f*(*v*_i_*_j_*, *v*_n_*_j_*)_calc_]. ^e^*L* (nm): NP length. ^f^PGA: phloroglucin aldehyde.

## Conclusion

N2D3Ss are a promising and plausible tool to help conventional NDDs cross the BBB. AI/ML algorithms can be instrumental in expediting the process of designing N2D3Ss. However, scientific literature lacks a sufficient number of real N2D3S experimental cases that characterize complex applications. In this context, the IFPTML model, encompassing both NDDs and NP models, could offer a practical solution. This approach has successfully addressed the challenges posed by the vast number of combinations of NP and NDD compounds and the wide range of conditions to be tested in N2D3S discovery. The results of the IFPTML-LDA and IFPTML-ANN techniques showed satisfactory performance, achieving Sp values of 73.0%–86.1% and Sn values of 70.0%–86.2% in the training and validation series, comprising 375,000 and 125,000 cases, respectively. Moreover, both models are easily accessible and provide logical solutions for predicting putative N2D3Ss. The most successful outcome was observed using non-linear models, specifically, the IFPTML-MLP model, which displayed Sn and Sp values of 85.8–86.2% and an AUROC value of 0.94 in the training and validation series. Furthermore, the analysis of three N2D3Ss samples yielded low SDV values, confirming the robustness of both IFPTML-LDA and IFPTML-ANN. In summary, the IFPTML models offer an initial solution for a rapid and less arduous pre-screening of putative N2D3Ss. This approach is widely utilized to minimize resource costs and save experimental time that would otherwise be spent on testing all possible combinations.

## Supporting Information

File 1 Detailed dataset information.

## Data Availability

The data generated and analyzed during this study is openly available in the Figshare repository at https://doi.org/10.6084/m9.figshare.25144544.

## References

[1] Chowdhury A, Kunjiappan S, Panneerselvam T, Somasundaram B, Bhattacharjee C (2017). Int Nano Lett.

[2] Murray C, Lopez A D (1994). Bull W H O.

[3] Zhang Q, Qian W-J, Knyushko T V, Clauss T R W, Purvine S O, Moore R J, Sacksteder C A, Chin M H, Smith D J, Camp D G (2007). J Proteome Res.

[4] Aslan M, Ozben T (2004). Curr Alzheimer Res.

[5] Hanumanthappa R, Venugopal D M, P C N, Shaikh A, B.M S, Heggannavar G B, Patil A A, Nanjaiah H, Suresh D, Kariduraganavar M Y (2023). ACS Omega.

[6] Contestabile A, Ciani E, Contestabile A (2008). Neurochem Res.

[7] Agnihotri T G, Jadhav G S, Sahu B, Jain A (2022). Drug Delivery Transl Res.

[8] Calzoni E, Cesaretti A, Polchi A, Di Michele A, Tancini B, Emiliani C (2019). J Funct Biomater.

[9] Polchi A, Magini A, Mazuryk J, Tancini B, Gapiński J, Patkowski A, Giovagnoli S, Emiliani C (2016). Nanomaterials.

[10] Diéguez-Santana K, González-Díaz H (2021). Nanoscale.

[11] Cacciatore I, Ciulla M, Fornasari E, Marinelli L, Di Stefano A (2016). Expert Opin Drug Delivery.

[12] Asefy Z, Hoseinnejhad S, Ceferov Z (2021). Neurol Sci.

[13] Shayganfard M (2022). Curr Pharm Biotechnol.

[14] Verma R, Sartaj A, Qizilbash F F, Ghoneim M M, Alshehri S, Imam S S, Kala C, Alam M S, Gilani S J, Taleuzzaman M (2022). Curr Drug Metab.

[15] Syed A A, Reza M I, Singh P, Thombre G K, Gayen J R (2021). Curr Drug Metab.

[16] Yu Y, O’Rourke A, Lin Y-H, Singh H, Eguez R V, Beyhan S, Nelson K E (2020). ACS Infect Dis.

[17] Pribut N, Kaiser T M, Wilson R J, Jecs E, Dentmon Z W, Pelly S C, Sharma S, Bartsch P W, Burger P B, Hwang S S (2020). ACS Infect Dis.

[18] Wang X, Perryman A L, Li S-G, Paget S D, Stratton T P, Lemenze A, Olson A J, Ekins S, Kumar P, Freundlich J S (2019). ACS Infect Dis.

[19] Cooper C J, Krishnamoorthy G, Wolloscheck D, Walker J K, Rybenkov V V, Parks J M, Zgurskaya H I (2018). ACS Infect Dis.

[20] Duncan G A, Bevan M A (2015). Nanoscale.

[21] Zhou H, Cao H, Matyunina L, Shelby M, Cassels L, McDonald J F, Skolnick J (2020). Mol Pharmaceutics.

[22] Sun L, Yang H, Cai Y, Li W, Liu G, Tang Y (2019). J Chem Inf Model.

[23] Kolesov A, Kamyshenkov D, Litovchenko M, Smekalova E, Golovizin A, Zhavoronkov A (2014). Comput Math Methods Med.

[24] Heider D, Senge R, Cheng W, Hüllermeier E (2013). Bioinformatics.

[25] Manganelli S, Leone C, Toropov A A, Toropova A P, Benfenati E (2016). Chemosphere.

[26] Toropova A P, Toropov A A, Rallo R, Leszczynska D, Leszczynski J (2015). Ecotoxicol Environ Saf.

[27] Toropova A P, Toropov A A, Veselinović A M, Veselinović J B, Benfenati E, Leszczynska D, Leszczynski J (2016). Ecotoxicol Environ Saf.

[28] Rybińska-Fryca A, Mikolajczyk A, Puzyn T (2020). Nanoscale.

[29] Le T C, Yin H, Chen R, Chen Y, Zhao L, Casey P S, Chen C, Winkler D A (2016). Small.

[30] Ahmadi S, Toropova A P, Toropov A A (2020). Nanotoxicology.

[31] Ojha P K, Kar S, Roy K, Leszczynski J (2019). Nanotoxicology.

[32] Sizochenko N, Gajewicz A, Leszczynski J, Puzyn T (2018). Nanoscale.

[33] Tasi D A, Csontos J, Nagy B, Kónya Z, Tasi G (2018). Nanoscale.

[34] Villaverde J J, Sevilla-Morán B, López-Goti C, Alonso-Prados J L, Sandín-España P (2018). Sci Total Environ.

[35] Sizochenko N, Leszczynska D, Leszczynski J (2017). Nanomaterials.

[36] Manganelli S, Benfenati E (2017). Methods Mol Biol (N Y, NY, U S).

[37] Puzyn T, Rasulev B, Gajewicz A, Hu X, Dasari T P, Michalkova A, Hwang H-M, Toropov A, Leszczynska D, Leszczynski J (2011). Nat Nanotechnol.

[38] Sizochenko N, Mikolajczyk A, Jagiello K, Puzyn T, Leszczynski J, Rasulev B (2018). Nanoscale.

[39] Toropov A A, Toropova A P, Benfenati E, Gini G, Puzyn T, Leszczynska D, Leszczynski J (2012). Chemosphere.

[40] Gonzalez-Diaz H, Arrasate S, Gomez-SanJuan A, Sotomayor N, Lete E, Besada-Porto L, Ruso J M (2013). Curr Top Med Chem.

[41] Alonso N, Caamaño O, Romero-Duran F J, Luan F, D. S. Cordeiro M N, Yañez M, González-Díaz H, García-Mera X (2013). ACS Chem Neurosci.

[42] Diez-Alarcia R, Yáñez-Pérez V, Muneta-Arrate I, Arrasate S, Lete E, Meana J J, González-Díaz H (2019). ACS Chem Neurosci.

[43] González-Díaz H, Riera-Fernández P, Pazos A, Munteanu C R (2013). BioSystems.

[44] González-Díaz H, Herrera-Ibatá D M, Duardo-Sánchez A, Munteanu C R, Orbegozo-Medina R A, Pazos A (2014). J Chem Inf Model.

[45] González-Díaz H, Riera-Fernández P (2012). J Chem Inf Model.

[46] Concu R, D. S. Cordeiro M N, Munteanu C R, González-Díaz H (2019). J Proteome Res.

[47] Martínez-Arzate S G, Tenorio-Borroto E, Barbabosa Pliego A, Díaz-Albiter H M, Vázquez-Chagoyán J C, González-Díaz H (2017). J Proteome Res.

[48] Quevedo-Tumailli V F, Ortega-Tenezaca B, González-Díaz H (2018). J Proteome Res.

[49] Santana R, Zuluaga R, Gañán P, Arrasate S, Onieva E, González-Díaz H (2020). Nanoscale.

[50] Romero Durán F J, Alonso N, Caamaño O, García-Mera X, Yañez M, Prado-Prado F J, González-Díaz H (2014). Int J Mol Sci.

[51] Luan F, Cordeiro M N D S, Alonso N, García-Mera X, Caamaño O, Romero-Duran F J, Yañez M, González-Díaz H (2013). Bioorg Med Chem.

[52] Gonzalez-Diaz H (2010). Curr Pharm Des.

[53] Kleandrova V V, Luan F, González-Díaz H, Ruso J M, Speck-Planche A, Cordeiro M N D S (2014). Environ Sci Technol.

[54] Luan F, Kleandrova V V, González-Díaz H, Ruso J M, Melo A, Speck-Planche A, Cordeiro M N D S (2014). Nanoscale.

[55] Santana R, Zuluaga R, Gañán P, Arrasate S, Onieva E, González-Díaz H (2019). Nanoscale.

[56] Santana R, Zuluaga R, Gañán P, Arrasate S, Onieva E, Montemore M M, González-Díaz H (2020). Mol Pharmaceutics.

[57] Urista D V, Carrué D B, Otero I, Arrasate S, Quevedo-Tumailli V F, Gestal M, González-Díaz H, Munteanu C R (2020). Biology (Basel, Switz).

[58] Romero-Durán F J, Alonso N, Yañez M, Caamaño O, García-Mera X, González-Díaz H (2016). Neuropharmacology.

[59] Ortega-Tenezaca B, González-Díaz H (2021). Nanoscale.

[60] Bento A P, Gaulton A, Hersey A, Bellis L J, Chambers J, Davies M, Krüger F A, Light Y, Mak L, McGlinchey S (2014). Nucleic Acids Res.

[61] Davies M, Nowotka M, Papadatos G, Dedman N, Gaulton A, Atkinson F, Bellis L, Overington J P (2015). Nucleic Acids Res.

[62] Gaulton A, Bellis L J, Bento A P, Chambers J, Davies M, Hersey A, Light Y, McGlinchey S, Michalovich D, Al-Lazikani B (2012). Nucleic Acids Res.

[63] Gusenbauer M, Haddaway N R (2020). Res Synth Methods.

[64] Lu Z (2011). Database.

[65] Islamaj Dogan R, Murray G C, Névéol A, Lu Z (2009). Database.

[66] Li Y, Li H, Pickard F C, Narayanan B, Sen F G, Chan M K Y, Sankaranarayanan S K R S, Brooks B R, Roux B (2017). J Chem Theory Comput.

[67] Xia R, Kais S (2018). Nat Commun.

[68] Na G S, Chang H, Kim H W (2020). Phys Chem Chem Phys.

[69] Concu R, Kleandrova V V, Speck-Planche A, Cordeiro M N D S (2017). Nanotoxicology.

[70] Kleandrova V V, Luan F, González-Díaz H, Ruso J M, Melo A, Speck-Planche A, Cordeiro M N D S (2014). Environ Int.

[71] Gaulton A, Hersey A, Nowotka M, Bento A P, Chambers J, Mendez D, Mutowo P, Atkinson F, Bellis L J, Cibrián-Uhalte E (2017). Nucleic Acids Res.

[72] Mendez D, Gaulton A, Bento A P, Chambers J, De Veij M, Félix E, Magariños M P, Mosquera J F, Mutowo P, Nowotka M (2019). Nucleic Acids Res.

[73] Moriwaki H, Tian Y-S, Kawashita N, Takagi T (2018). J Cheminf.

[74] Hill T, Lewicki P (2006). Statistics: Methods and Applications.

[75] Bendel R B, Afifi A A (1977). J Am Stat Assoc.

[76] Gamberger D, Lavrac N (2002). J Artif Intell Res.

[77] Huberty C J, Olejnik S (2006). Applied MANOVA and discriminant analysis.

[78] Hanczar B, Hua J, Sima C, Weinstein J, Bittner M, Dougherty E R (2010). Bioinformatics.

[79] Bian L, Sorescu D C, Chen L, White D L, Burkert S C, Khalifa Y, Zhang Z, Sejdic E, Star A (2019). ACS Appl Mater Interfaces.

[80] Alafeef M, Srivastava I, Pan D (2020). ACS Sens.

[81] Sun B, Fernandez M, Barnard A S (2017). J Chem Inf Model.

[82] Barnard A S, Opletal G (2019). Nanoscale.

[83] He J, He C, Zheng C, Wang Q, Ye J (2019). Nanoscale.

[84] Yan T, Sun B, Barnard A S (2018). Nanoscale.

[85] Speck-Planche A, Kleandrova V V, Luan F, Cordeiro M N D S (2015). Nanomedicine (London, U K).

[86] Nocedo-Mena D, Cornelio C, Camacho-Corona M d R, Garza-González E, Waksman de Torres N, Arrasate S, Sotomayor N, Lete E, González-Díaz H (2019). J Chem Inf Model.

[87] Leys C, Ley C, Klein O, Bernard P, Licata L (2013). J Exp Soc Psychol.

[88] Arnedo M (1999). Bol Soc Entomol Aragonesa.

[89] Hitchcock S A, Pennington L D (2006). J Med Chem.

[90] Doan K M M, Humphreys J E, Webster L O, Wring S A, Shampine L J, Serabjit-Singh C J, Adkison K K, Polli J W (2002). J Pharmacol Exp Ther.

[91] Chithrani B D, Ghazani A A, Chan W C W (2006). Nano Lett.

[92] Shilo M, Sharon A, Baranes K, Motiei M, Lellouche J-P M, Popovtzer R (2015). J Nanobiotechnol.

